# A prognostic nomogram model for non-complete remission following initial radioiodine therapy in Graves’ hyperthyroidism

**DOI:** 10.3389/fendo.2025.1692702

**Published:** 2025-10-28

**Authors:** Congcong Wang, Yutian Li, Guohua Qin, Yanhui Song, Xue Yang, Yaqi Lu, Xufu Wang

**Affiliations:** ^1^ Department of Nuclear Medicine, The Affiliated Hospital of Qingdao University, Qingdao, Shandong, China; ^2^ Department of Radiology, Qingdao Women and Children’s Hospital, Qingdao, Shandong, China; ^3^ Department of Medical Oncology, The Affiliated Hospital of Qingdao University, Qingdao, Shandong, China

**Keywords:** Graves’ hyperthyroidism, radioiodine therapy, free triiodothyronine change, non-complete remission, nomogram model

## Abstract

**Background:**

Radioiodine (RAI) therapy, while established for Graves’ hyperthyroidism (GH), exhibits variable efficacy (50-80% cure rates), with non-complete remission (NCR) necessitating retreatment. In the study, we aimed to identify independent predictors of NCR and develop a validated nomogram for personalized RAI outcome prediction.

**Methods:**

Data from 285 GH patients undergoing initial RAI therapy were retrospectively analyzed and randomly allocated into training (n=199) and validation (n=86) cohorts at a 7:3 ratio. Univariate followed by multivariate logistic regression identified independent predictors of NCR in the training cohort. These variables informed the construction of a prognostic nomogram model, subsequently verified in the validation cohort through calibration, receiver operating characteristic (ROC) curve, and decision curve analysis (DCA) to assess model reliability, discriminative ability, and clinical utility.

**Results:**

Thyroid mass (TM), 24-hour RAI uptake (RAIU24h), effective half-life (Teff), and free triiodothyronine reduction at 1-month post-therapy (ΔFT3) were independent predictors. The prognostic nomogram integrating these variables exhibited superior discriminative performance in both training (AUC = 0.919) and validation cohorts (AUC = 0.901). Calibration curves confirmed high fidelity between predicted and observed NCR probabilities. DCA demonstrated significant clinical net benefit across threshold probabilities.

**Conclusion:**

TM, RAIU24h, Teff, and ΔFT3 are critical determinants of RAI efficacy in GH. The validated nomogram enables precise NCR risk stratification, facilitating optimized activity prescription to improve remission rates.

## 1 Introduction

Graves’ hyperthyroidism (GH), an organ-specific autoimmune disorder, represents the most prevalent cause of thyrotoxicosis, characterized by excessive thyroid hormone production due to thyroid-stimulating hormone (TSH) receptor antibodies (TRAb) (1–3). The clinical triad of hypermetabolism, diffuse goiter, and orbitopathy stems from systemic effects of thyroid hormone excess, which also disrupts bone metabolism, cardiovascular function, and other physiological processes. Left untreated, GH carries significant morbidity risks including atrial fibrillation, osteoporosis, and thyrotoxic crisis (4–6).

The current first-line therapeutic approaches for GH include antithyroid drugs (ATDs), radioiodine (RAI) therapy, and thyroidectomy. It should be noted that these treatments primarily aim to reduce circulating thyroid hormone levels rather than directly addressing the underlying etiology, such as modulating TRAb or correcting thyroid autoimmune dysregulation (4, 5).

RAI therapy has been established as a treatment modality for hyperthyroidism for over eight decades. Recent epidemiological studies indicate that 59% of North American clinicians prefer RAI as the initial treatment for GH (7). Compared with alternative therapies, RAI offers distinct advantages including superior efficacy, fewer adverse effects, and more rapid onset of therapeutic action. Current clinical guidelines strongly recommend RAI for patients who exhibit poor response to ATDs, experience disease recurrence after ATDs withdrawal, develop ATDs-related adverse reactions (e.g., leukopenia or hepatic dysfunction), or present with complications such as atrial fibrillation (4, 5). Despite the well-established efficacy of RAI, treatment outcomes exhibit considerable variability, with single-dose cure rates ranging from 50-80% across studies, and hypothyroidism developing in 20-30% of patients within the first year (8–10).

Existing predictive models for RAI therapy outcomes in GH have largely relied on static pre-therapeutic parameters, such as thyroid mass (TM), 24-hour radioiodine uptake (RAIU24h), and effective half-life (Teff) (9, 11, 12). A significant limitation of these models is their inability to account for early biological response, which reflects the initial radiation-induced thyroid follicular cell damage. The dynamics of thyroid hormone levels, particularly the reduction in free triiodothyronine (FT3)/free thyroxine (FT4) at one month post-RAI, offer a pathophysiologically grounded biomarker that may serve as an early indicator of treatment efficacy. However, the prognostic value of this early hormonal response for predicting non-remission remains inadequately characterized. Therefore, this study was designed to elucidate the predictive power of early thyroid function dynamics and to identify determinants of treatment non-remission, with the ultimate goal of informing personalized therapeutic strategies.

## 2 Materials and methods

### 2.1 Study conduct and patients

This retrospective study was conducted at the Affiliated Hospital of Qingdao University. The protocol was approved by the institution’s Ethics Review Board with a waiver of informed consent (Ethics approval number: QYFY WZLL 30462). From January 2021 to December 2023, all GH patients who first underwent RAI therapy in the Affiliated Hospital of Qingdao University were reviewed.

GH was diagnosed according to established criteria comprising: (i) biochemical confirmation of thyrotoxicosis (suppressed TSH with elevated free thyroid hormones), (ii) diffuse thyroid enlargement on palpation, and (iii) either TRAb positivity or increased radioactive iodine uptake (RAIU). Characteristic extrathyroidal manifestations (orbitopathy or pretibial myxedema) provided supporting evidence when present.

The inclusion criteria were as follows: (i) confirmed GH diagnosis; (ii) discontinuation of ATDs for at least one week preceding RAI, and (iii) no ATDs administration following RAI. The exclusion criteria comprised: (i) previous or fractionated RAI, (ii) lost to follow-up or incomplete clinical data, and (iii) concurrent suspicious malignant thyroid nodules.

### 2.2 Data collection and RAI procedures

Prior to RAI administration, all patients were instructed to maintain a low-iodine diet and discontinue iodine-containing medications for 7–14 days. ATDs were discontinued as recommended by the 2016 ATA hyperthyroidism guidelines (methimazole for 3–5 days or propylthiouracil for 1–2 weeks) prior to RAI administration to enhance thyroidal RAI uptake (5). Pregnancy and lactation women constituted absolute contraindications for treatment (5, 13).

Laboratory assessments were performed to evaluate thyroid function, including TSH (reference range: 0.75-5.6 μIU/mL), free triiodothyronine (FT3; reference range: 3.1-6.8 pmol/L), free thyroxine (FT4; reference range: 12.8-21.3 pmol/L), thyroglobulin antibody (TgAb; reference range: 0–115 IU/mL), thyroid peroxidase antibody (TPOAb; reference range: 0–34 IU/mL), and TRAb (reference range: 0-1.75 IU/mL). Serum levels of FT3, FT4, TSH, TgAb, TPOAb, and TRAb were quantified using a Cobas e 801 automated electrochemiluminescence immunoassay analyzer (Roche Diagnostics GmbH, Mannheim, Germany) with the corresponding reagent kits (Elecsys FT3 III, Elecsys FT4 III, Elecsys TSH, Elecsys Anti-Tg, Elecsys Anti-TPO, and Elecsys Anti-TSHR), in strict accordance with the manufacturer’s protocols. All patients routinely underwent thyroid ultrasonography to assess for the presence of nodules, as well as ^99m^Tc-pertechnetate thyroid scan to exclude subacute thyroiditis and measure thyroid volume. Thyroid volume (TV) was calculated using the following formula: TV (cm³)= mean thyroid lobe height (cm)×sum of bilateral frontal projected areas (cm²)×K, where K is a correction factor ranging between 0.23 and 0.32, depending on imaging conditions (14). Subsequently, TM was estimated from the volume by assuming a tissue density of 1.0 g/cm³ [TM (g)=1×TV (cm³)] (13, 15). The thyroid RAIU was dynamically assessed at 2, 4, and 24 hours post-administration to determine the RAIU24h and Teff.

The therapeutic ^131^I activity was calculated based on TM and RAIU24h using the following formula: Oral ^131^I activity (μCi) = [prescribed dose (μCi/g) × TM (g)]/RAIU24h (%). Three experienced nuclear medicine physicians determined the prescribed iodine dose per gram of thyroid tissue for each patient according to their clinical condition, typically ranging between 70-150 μCi/g.

We conducted a comprehensive review of clinical parameters obtained from electronic medical records, including: patient demographics (age, gender, disease course, TM, body mass index [BMI]); thyroid function tests (FT3, FT4, TSH); immunological markers (TRAb, TgAb, TPOAb); nuclear medicine parameters (RAIU24h, Teff); and treatment-related data (total RAI dose). Teff was calculated from the serial RAI uptake measurements. It is defined as the time required for the RAI activity within the thyroid gland to decay to 50% of its initial value, resulting from the combined effects of physical decay and biological clearance (5, 9).

For analytical purposes, continuous variables of thyroid autoantibodies were categorized based on the assay’s reference ranges and established clinical cut-off points. Specifically, TRAb levels were classified as follows: <1.75 IU/mL (normal), 1.75–40 IU/mL (mildly elevated), and >40 IU/mL (significantly elevated). TgAb levels were categorized as <115 IU/mL (normal), 115–4000 IU/mL (moderately elevated), and >4000 IU/mL (highly elevated). TPOAb levels were categorized as <34 IU/mL (normal), 34–600 IU/mL (moderately elevated), and >600 IU/mL (highly elevated).

### 2.3 Magnitude of thyroid function changes at one month post-RAI

FT3 and FT4 levels were measured at two time points: 1–2 days before RAI and 1 month after RAI, designated as pre-RAI FT3, post-RAI FT3, pre-RAI FT4, and post-RAI FT4, respectively. The percent changes in thyroid hormone levels (ΔFT3 and ΔFT4) at 1 month post-RAI were calculated using the following equations: ΔFT3 = [(pre-RAI FT3 - post-RAI FT3)/pre-RAI FT3] × 100%; ΔFT4 = [(pre-RAI FT4 - post-RAI FT4)/pre-RAI FT4] × 100%.

### 2.4 Evaluation for the therapeutic efficacy of RAI

Following RAI therapy, all enrolled patients underwent a comprehensive clinical and biochemical evaluation during follow-up visits conducted at 6 to 12 months after treatment. Therapeutic outcomes were rigorously classified based on predefined response criteria (4, 5):

1. Euthyroidism: Resolution of GH symptoms with normalization of FT3, FT4, and TSH levels;
2. Hypothyroidism: Presence of hypothyroid symptoms (which could be absent in subclinical cases) accompanied by subnormal FT3 or FT4 and elevated TSH concentrations.
3. Partial Remission: Improvement in GH-related symptoms and signs with partial reduction, but not normalization of thyroid hormone levels.
4. Non-remission: Persistent or worsening hyperthyroid manifestations without significant decrease in FT3/FT4 concentrations.

For analytical purposes, patients achieving either euthyroidism or hypothyroidism were classified as the “complete remission” (CR) group, while those with persistent hyperthyroidism (including cases of partial remission and treatment failure) were categorized as the “non-complete remission” (NCR) group (9, 11, 12).

### 2.5 Study design and statistical analysis

GH patients were randomly allocated into training and cohorts at a 7:3 ratio. Statistical analysis was performed to evaluate the differences in ΔFT3 and ΔFT4 values between treatment response groups within the training cohort using the Mann-Whitney U test. Additionally, receiver operating characteristic (ROC) curve analysis was conducted to assess the predictive performance of these parameters for NCR. Univariate logistic regression analysis was initially performed in the training cohort to identify potential predictors of RAI response, followed by multivariate analysis to determine independent risk factors for NCR. These significant predictors were subsequently incorporated into a prognostic nomogram constructed to estimate the probability of NCR following RAI therapy. The predictive accuracy and discriminative ability of the nomogram were then rigorously evaluated in the validation cohort.

The nomogram’s discriminative ability was evaluated using Harrell’s concordance index (C-index), which measures the agreement between predicted probabilities and actual outcomes. ROC curve analysis further assessed predictive performance, with the area under the curve (AUC) serving as a quantitative measure of accuracy. Calibration curves graphically compared predicted probabilities against observed event rates, evaluating model precision. Additionally, decision curve analysis (DCA) determined clinical utility by quantifying net benefit across various probability thresholds. All statistical analyses—including nomogram construction (rms[6.4.0], ResourceSelection[0.3-5]), C-index calculation (rms[6.4.0], ResourceSelection[0.3-5]), ROC analysis (pROC[1.18.0], ggplot2[3.4.4]), calibration curve assessment (rms[6.4.0], ResourceSelection[0.3-5]), and DCA evaluation (rmda[1.6], ggplot2[3.4.4])—were performed using R software (version 4.2.1). A two-tailed P-value < 0.05 defined statistical significance.

## 3 Results

### 3.1 Baseline clinical characteristics

**Figure 1:**
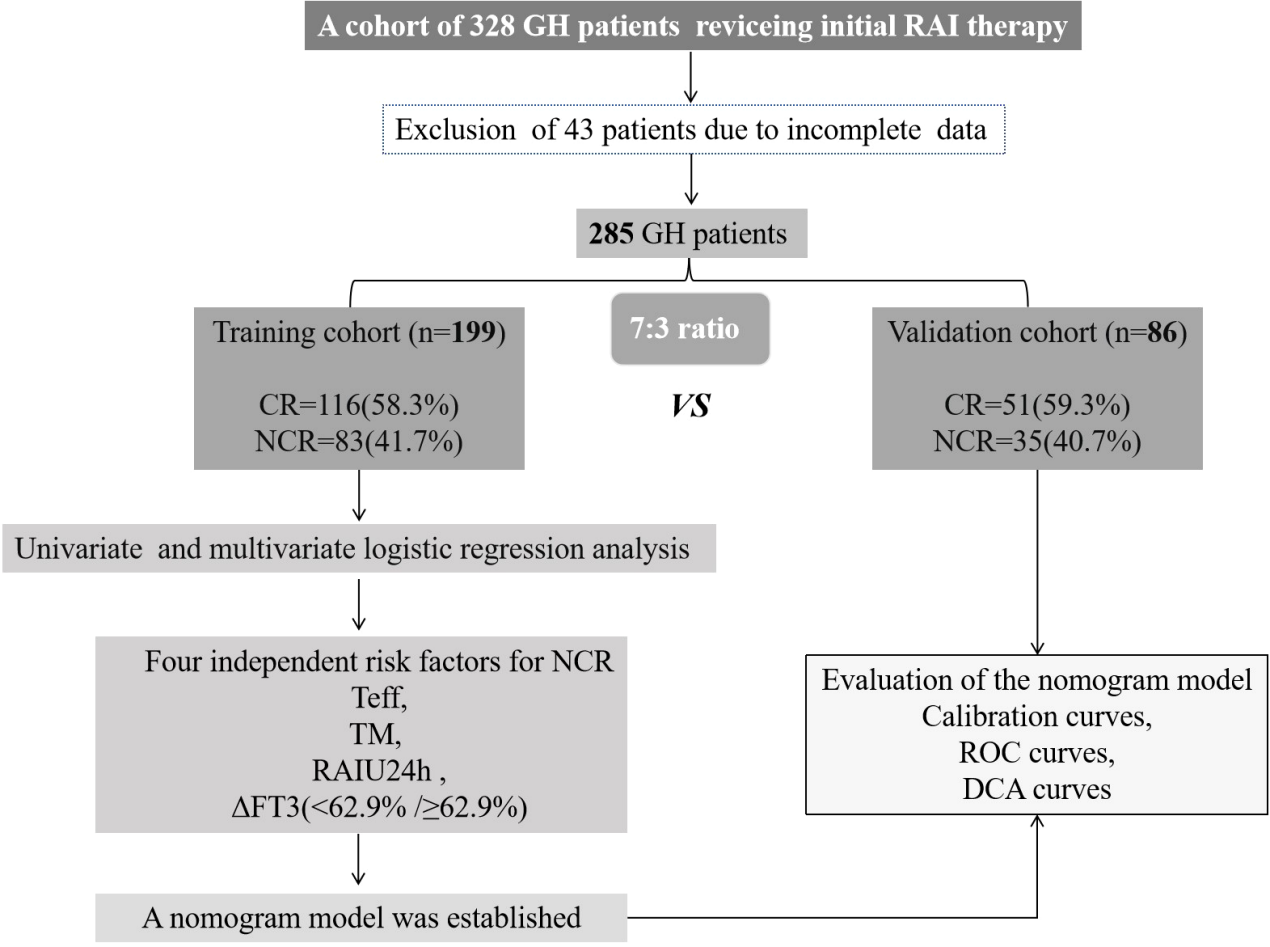
Research flowchart. GH, Graves’ hyperthyroidism; RAI, radioiodine; CR, complete remission; NCR, non-complete remission; Teff, thyroid effective half-life; TM, thyroid mass; RAIU24h, 24-hour radioactive iodine uptake; ROC, receiver operating characteristic; and DCA, decision curve analysis.

Between January 2021 and December 2023, our institution enrolled 328 GH patients for RAI therapy. Following the study protocol, 43 subjects were excluded due to loss to follow-up or incomplete clinical data, yielding an 86.9% (285/328) eligibility rate for final analysis. The cohort was subsequently randomized into training (n=199) and validation (n=86) subsets at a 7:3 ratio for predictive model development and therapeutic outcome evaluation, as detailed in **Figure 1**.

Baseline clinical characteristics of the GH patients were well-balanced between the training and validation cohorts, as summarized in **Table 1** (all p>0.05).

**Table 1:** Clinicopathologic characteristics of 285 GH patients.

Characteristics	Cohort, no. (%)		P value
	Training (n = 199)	Validation (n = 86)	
Gender			0.166
Female	152 (53.3%)	72 (25.3%)	
Male	47 (16.5%)	14 (4.9%)	
Age (years)	41 (30, 56)	39 (29, 52)	0.525
BMI (kg/m^2^)			0.285
BMI<18.5	26 (9.1%)	14 (4.9%)	
18.5≤BMI<24.9	115 (40.4%)	55 (19.3%)	
25≤BMI<29.9	45 (15.8%)	11 (3.9%)	
30≤BMI	13 (4.6%)	6 (2.1%)	
Disease course			0.564
≤2 years	67 (23.5%)	32 (11.2%)	
>2 years	132 (46.3%)	54 (18.9%)	
TRAb (IU/mL)			0.974
TRAb <1.75	8 (2.8%)	3 (1.1%)	
1.75≤TRAb ≤ 40	158 (55.4%)	69 (24.2%)	
TRAb>40	33 (11.6%)	14 (4.9%)	
TgAb (IU/mL)			0.376
TgAb <115	95 (33.3%)	44 (15.4%)	
115≤TgAb ≤ 4000	85 (29.8%)	38 (13.3%)	
TgAb>4000	19 (6.7%)	4 (1.4%)	
TPOAb (IU/mL)			0.456
TPOAb <34	46 (16.1%)	23 (8.1%)	
34≤TPOAb ≤ 600	108 (37.9%)	49 (17.2%)	
TPOAb>600	45 (15.8%)	14 (4.9%)	
Teff (days)	6.2 (5.1, 6.5)	6.3 (5.4, 6.8)	0.056
Total dose (mCi)	12.0 (9.0, 18.0)	14.0 (8.3, 20.8)	0.568
TM (g)	64.1 (47.5, 95.7)	68.5 (43.1, 97.9)	0.910
RAIU24h (%)	69.5 (60, 76.3)	67.5 (61.9, 77.5)	0.879
ΔFT3 (%)	61.5 (41.7, 74.5)	60.9 (38.1, 74.7)	0.774
ΔFT4 (%)	46.7 (30.0, 62.9)	47.2 (31.2, 67.2)	0.862

GH, Graves’ hyperthyroidism; BMI, body mass index; TRAb, thyroid-stimulating hormone receptor antibodies; TgAb, thyroglobulin antibody; TPOAb, thyroid peroxidase antibody; Teff, thyroid effective half-life; TM, thyroid mass; RAIU24h, 24-hour radioactive iodine uptake.

### 3.2 Clinical outcomes of GH patients after initial RAI therapy in the training and validation cohorts

**Figure 2:**
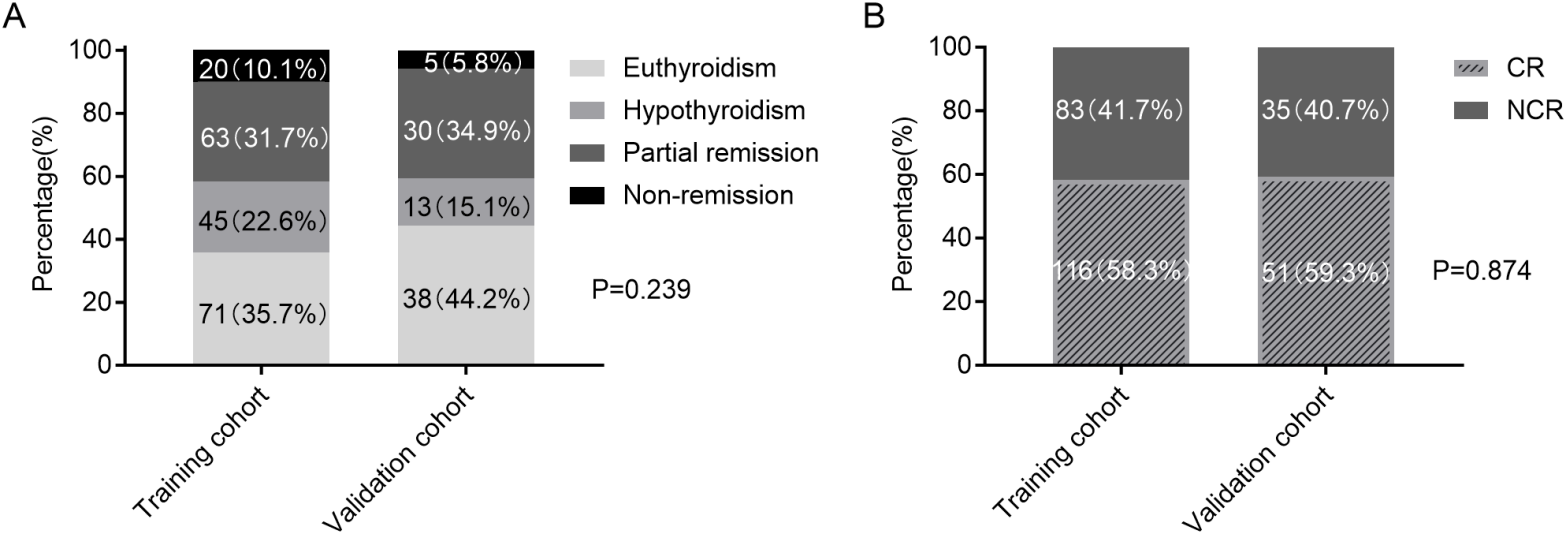
Clinical outcomes following RAI therapy in the training and validation cohorts. **(A)** Four-category therapeutic response (euthyroidism, hypothyroidism, partial remission, and non-remission). **(B)** Binary therapeutic response classification (CR *vs*. NCR). RAI, radioiodine; CR, complete remission; NCR, non-complete remission.

**Figure 2** demonstrated the clinical outcomes following initial RAI administration in the training and validation cohorts. NCR rates were similar between two cohorts (41.7% [83/199] *vs* 40.7% [35/86], p=0.874).

### 3.3 Comparison of the ΔFT3 and ΔFT4 in different therapeutic outcomes in the training cohort

**Figure 3:**
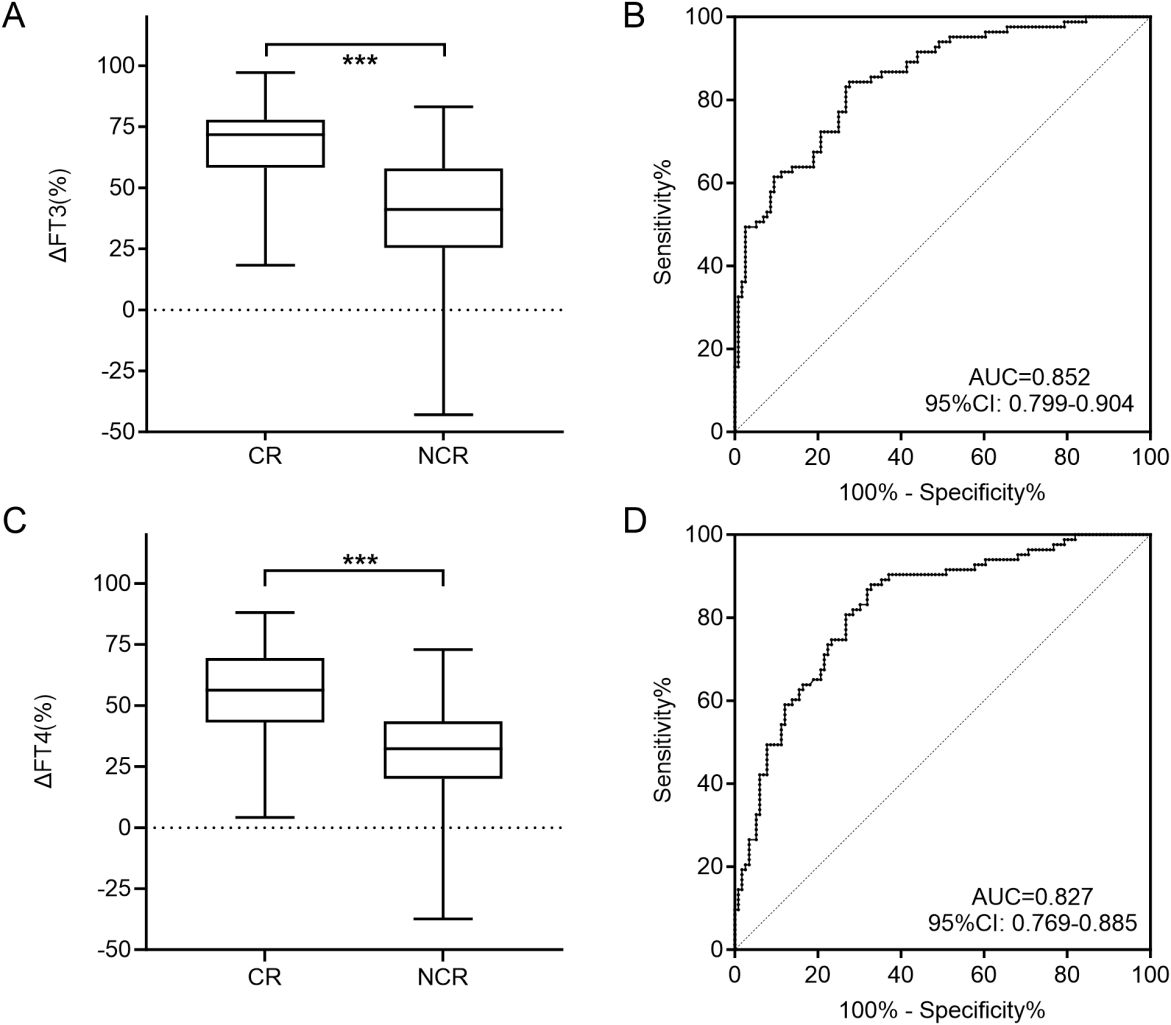
Comparison of the ΔFT3 **(A)** and ΔFT4 **(C)** level for CR and NCR in the training cohort. ROC curves of ΔFT3 **(B)** and ΔFT4 **(D)** for detecting NCR in the training cohort. CR, complete remission; NCR, non-complete remission; ROC, receiver operating characteristic; AUC, area under the ROC curve; *** P<0.001.

The median ΔFT3 in CR group was 71.8%, while that in the NCR group was only 41.1%. There was a statistical difference between the two groups (*U* = 1429.0, P < 0.001; **Figure 3A**). Meanwhile, the median ΔFT4 in CR group was 56.3%, which was also significantly higher than the 32.3% in the NCR group (*U* = 1667.0, P < 0.001; **Figure 3C**).

The cut-off value of ΔFT3 at 62.9% and ΔFT4 at 50.5% were obtained by ROC curve analyses to best distinguish CR and NCR, with corresponding specificities of ΔFT3 and ΔFT4 separately of 72.4% and 67.2%, and sensitivities of 84.3% and 88.0%, and AUCs of 0.852 and 0.827, respectively (**Figures 3B, D**). Thus, if the ΔFT3 was lower than 62.9% and/or ΔFT4 was lower than 50.5%, the curative effect was more likely to be NCR.

### 3.4 Risk factors for NCR of GH patients after initial RAI therapy in the training cohort

Upon analyzing the relation between clinical characteristics and NCR in GH patients after initial RAI therapy, 13 factors were included in the univariate analyses (**Table 2**). The results indicated that patients with disease course>2 years (P = 0.007), higher TRAb level (P = 0.002), lower TPOAb (P = 0.039), shorter Teff (P<0.001), lower RAIU24h (P<0.001), higher total dose (P<0.001), higher TM (P<0.001), ΔFT3 <62.9% (P<0.001) or ΔFT4 <50.5% (P<0.001) had a high likelihood of developing NCR. By contrast, no significant differences were found in gender (P = 0.636), age (P = 0.587), BMI (P = 0.051), and TgAb levels (P = 0.135).

**Table 2:** Univariate analyses for NCR in GH patients in the training cohort.

Characteristics	n	NCR group	CR group	*χ^2^/U*	*P*-value
Gender				0.224^a^	0.636
Female	152	62(40.8%)	90(59.2%)		
Male	47	21(44.7%)	26(55.3%)		
Age (years)		38(29, 54)	43.5(30, 57)	4596.5^b^	0.587
BMI (kg/m^2^)				7.763^a^	0.051
BMI<18.5	26	9(34.6%)	17(65.4%)		
18.5≤BMI<24.9	115	52(45.2%)	63(54.8%)		
25≤BMI<29.9	45	21(46.7%)	24(53.3%)		
30≤BMI	13	1(7.7%)	12(92.3%)		
Disease course				7.405^a^	0.007
≤2 years	67	19(28.4%)	48(71.6%)		
>2 years	132	64(48.5%)	68(51.5%)		
TRAb (IU/mL)				12.687^a^	0.002
TRAb <1.75	8	3(37.5%)	5(62.5%)		
1.75≤TRAb ≤ 40	158	57(36.1%)	101(63.9%)		
TRAb>40	33	23(69.7%)	10(30.3%)		
TgAb (IU/mL)				4.000^a^	0.135
TgAb <115	95	38(40.0%)	57(60.0%)		
115≤TgAb ≤ 4000	85	33(38.8%)	52(61.2%)		
TgAb>4000	19	12(63.2%)	7(36.8%)		
TPOAb (IU/mL)				6.464^a^	0.039
TPOAb <34	46	12(26.1%)	34(73.9%)		
34≤TPOAb ≤ 600	108	52(48.1%)	56(51.9%)		
TPOAb>600	45	19(42.2%)	26(57.8%)		
Teff (days)		5.2(4.3, 6.3)	6.4(5.7, 6.9)	2378.0^b^	<0.001
Total dose (mCi)		17.0(12.0, 28.0)	10.0(8.0, 14.0)	2228.5^b^	<0.001
TM (g)		95.9(61.3, 142.1)	55.1(42.8, 70.2)	2233.5^b^	<0.001
RAIU24h (%)		65.2(53.9, 74.8)	70.9(64.8, 78.6)	3291.0^b^	<0.001
ΔFT3 (%)				62.369^a^	<0.001
ΔFT3 <62.9%	102	70(68.6%)	32(31.4%)		
ΔFT3 ≥62.9%	97	13(13.4%)	84(86.6%)		
ΔFT4 (%)				59.752^a^	<0.001
ΔFT4 <50.5%	111	73(65.8%)	38(34.2%)		
ΔFT4 ≥50.5%	88	10(11.4%)	78(88.6%)		

NCR, non-complete remission; CR, complete remission; GH, Graves’ hyperthyroidism; BMI, body mass index; TRAb, thyroid-stimulating hormone receptor antibodies; TgAb, thyroglobulin antibody; TPOAb, thyroid peroxidase antibody; Teff, thyroid effective half-life; TM, thyroid mass; RAIU24h, 24-hour radioactive iodine uptake; ^a^means chi-squared test; ^b^means Mann-Whitney *U* test.

Multivariate analysis revealed that Teff (odds ratio [OR]: 0.438, 95% confidence interval [CI]: 0.288-0.667, P < 0.001), TM (OR: 1.031, 95% CI: 1.016-1.046, P < 0.001), RAIU24h (OR: 0.934, 95% CI: 0.899-0.969, P < 0.001), and ΔFT3(<62.9%/≥62.9%) (OR: 0.092, 95% CI: 0.037-0.225, P < 0.001) were significantly associated with the risk of NCR (**Table 3**).

**Table 3:** Multivariate analysis for NCR in GH patients using logistic regression in the training cohort.

Characteristics	P-value	OR	95%CI
Teff (days)	<0.001	0.438	0.288-0.667
TM (g)	<0.001	1.031	1.016-1.046
RAIU24h (%)	<0.001	0.934	0.899-0.969
ΔFT3(<62.9%/≥62.9%)	<0.001	0.092	0.037-0.225

NCR, non-complete remission; GH, Graves’ hyperthyroidism; Teff, thyroid effective half-life; TM, thyroid mass; RAIU24h, 24-hour radioactive iodine uptake; OR, odds ratio; CI, confidence interval.

### 3.5 Development of a prognostic nomogram for NCR rates in GH patients after initial RAI in the training cohort

**Figure 4:**
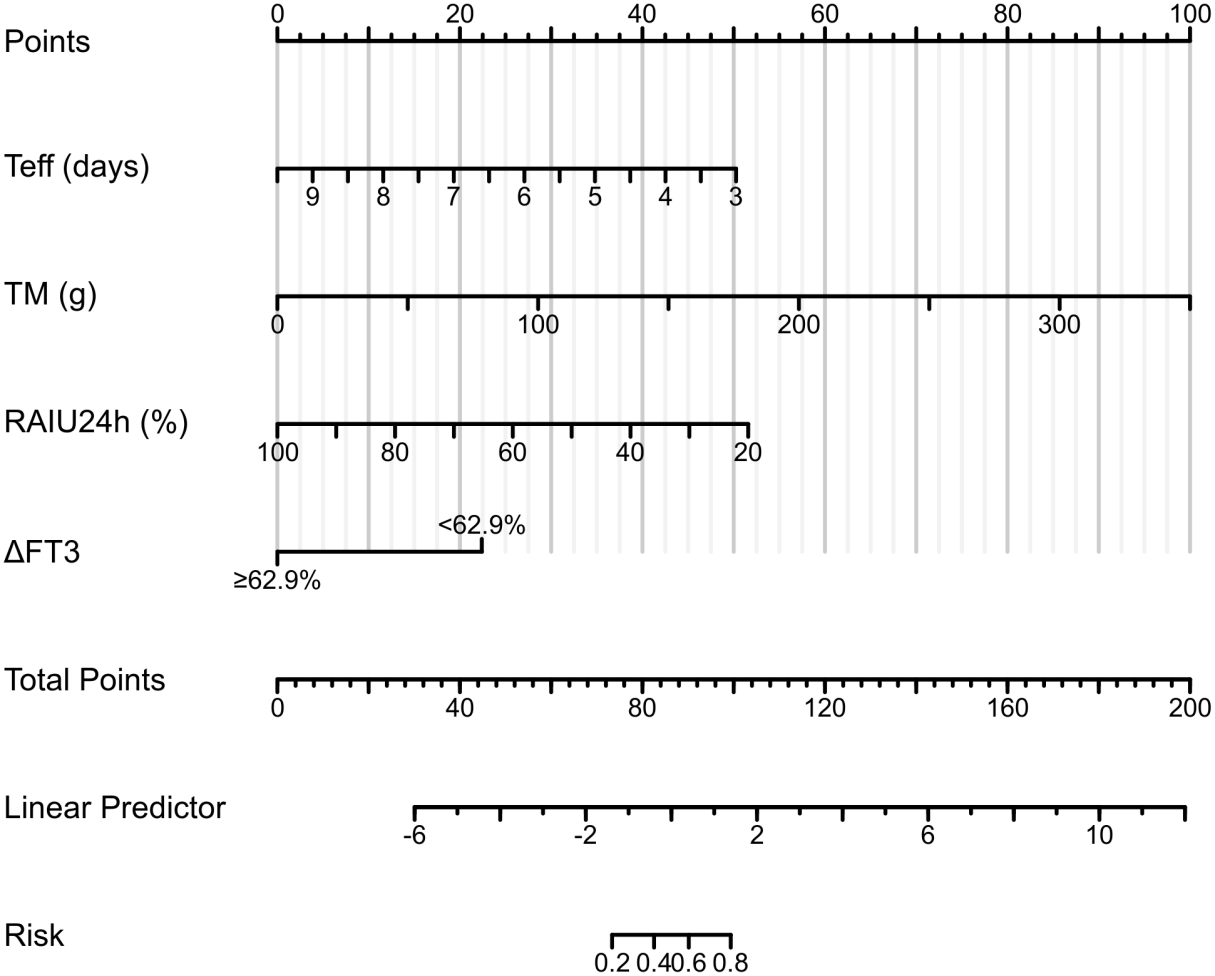
Clinical nomogram for predicting NCR risk following RAI therapy in the training cohort. Implementation protocol: To use it, one first locates a patient’s specific values for Teff, TM, and RAIU24h on their respective axes and draws a vertical line upward to the ‘Points’ axis to assign a partial score for each variable; for the categorical variable ΔFT3, one selects the corresponding point value based on whether the value is above or below the 62.9% threshold. The points for all four variables are then summed, and this total is located on the ‘Total Points’ axis; finally, by projecting a line downward from the total points to the bottom ‘Predicted Probability of NCR’ axis, the clinician can read the patient’s individualized risk of NCR, thereby facilitating personalized therapeutic decisions. NCR, non-complete remission; RAI, radioiodine; Teff, thyroid effective half-life; TM, thyroid mass; RAIU24h, 24-hour radioactive iodine uptake.

Based on the independent risk factors identified by multivariate logistic regression, we constructed a Nomogram to predict the probability of NCR in GH patients after initial RAI therapy in the training cohort. The predictive model incorporates weighted scores for each variable, with TM demonstrating the strongest association with NCR risk, followed by RAIU24h, Teff, and ΔFT3 (**Figure 4**). By summing the individual scores and projecting the total points onto the risk axis, clinicians can estimate the probability of NCR.

### 3.6 Evaluation of the nomogram model

**Figure 5:**
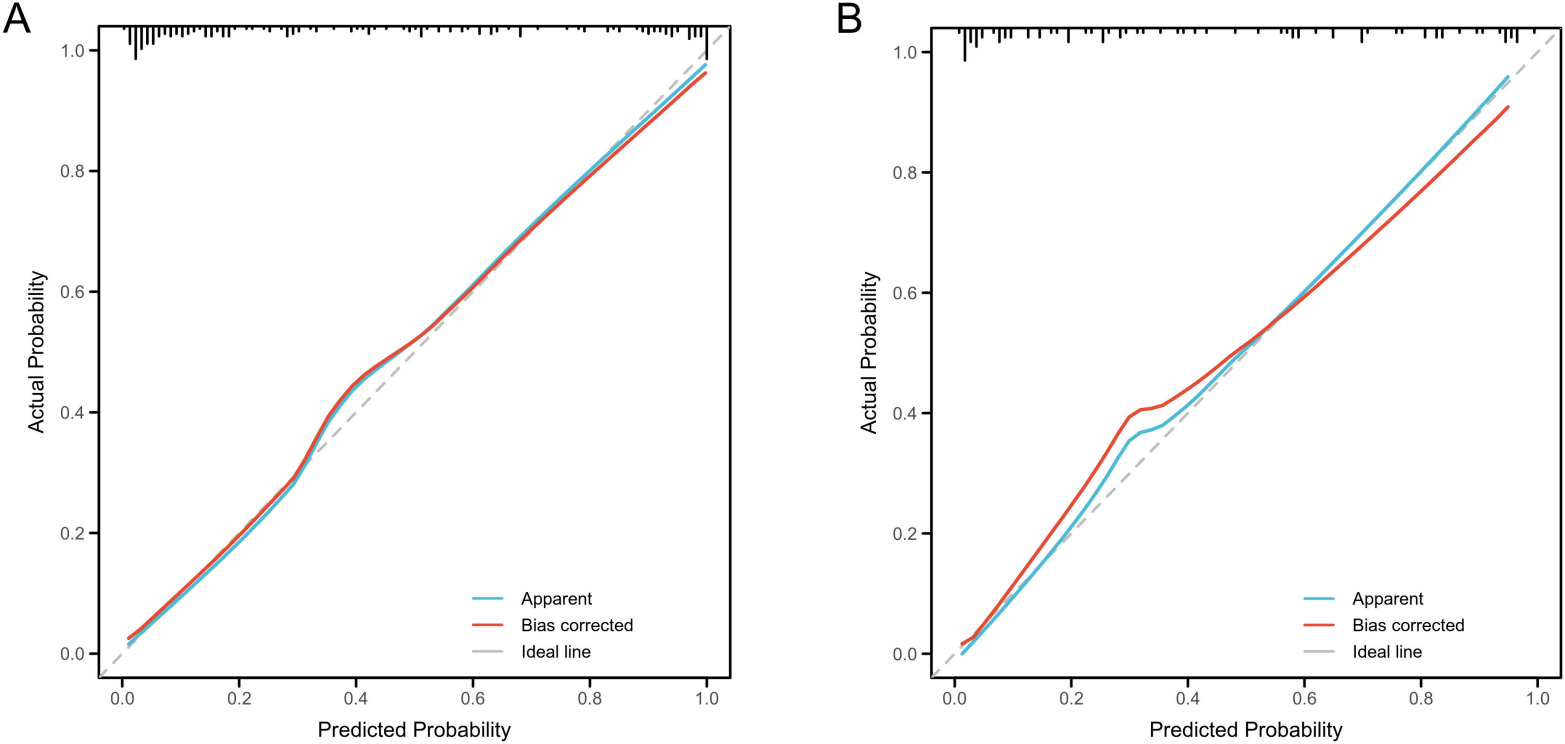
Performance evaluation of the nomogram for predicting non-complete remission (NCR) rates in training and validation cohorts. Calibration plots reveal strong agreement between predicted and observed NCR rates, with curves closely following the ideal reference line (45°diagonal) for both training **(A)** and validation **(B)** cohorts.

**Figure 6:**
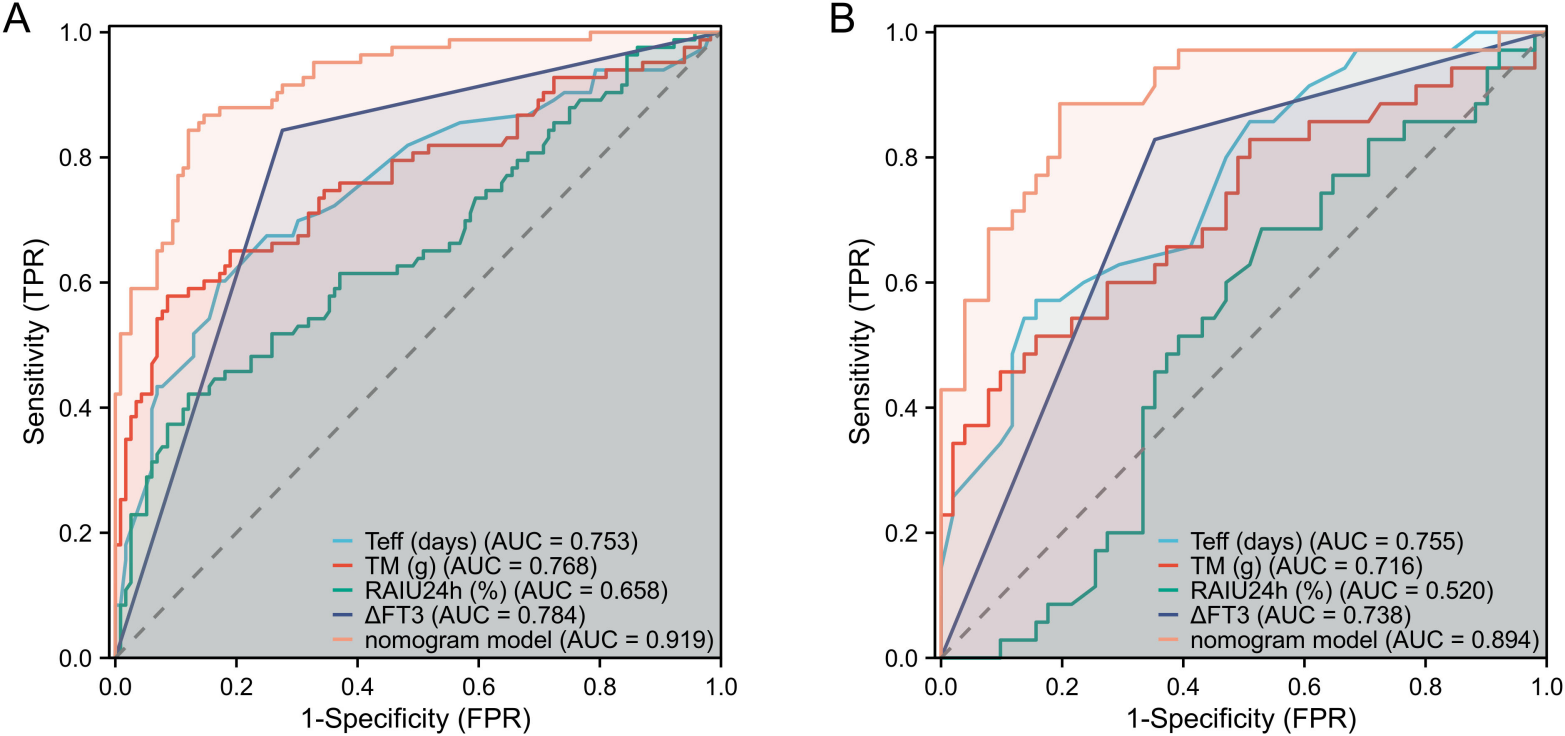
Performance evaluation of the nomogram for predicting NCR rates in training and validation cohorts. ROC curves demonstrate discrimination accuracy for NCR probability in both training **(A)** and validation **(B)** cohorts (AUC 0.919 and 0.894, respectively). NCR, non-complete remission; AUC, area under the ROC curve.

**Figure 7:**
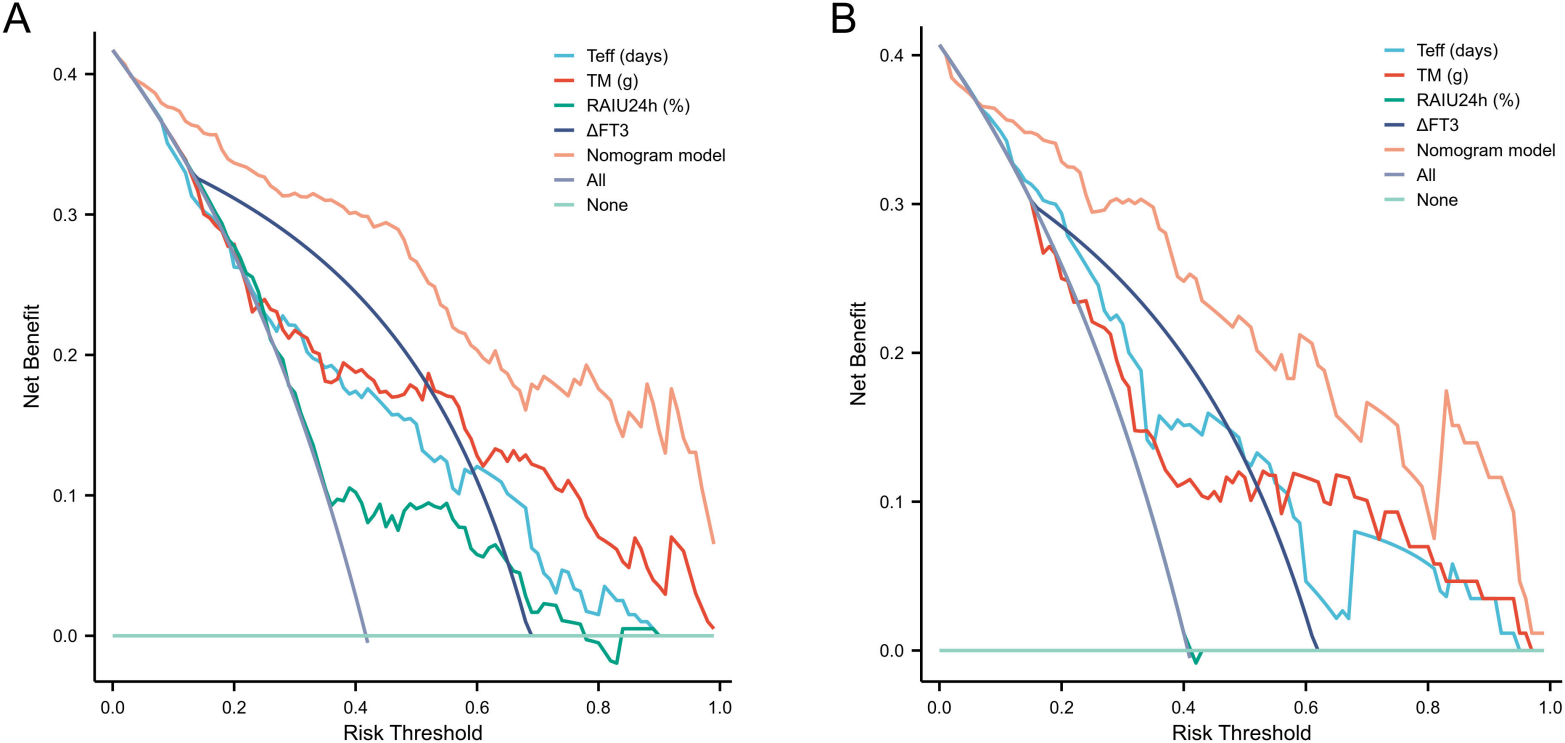
Performance evaluation of the nomogram for predicting NCR rates in training and validation cohorts. DCA confirms clinical utility, showing significant net benefit across for therapeutic decision-making in both training **(A)** and validation **(B)** cohorts. NCR, non-complete remission; DCA, decision curve analysis.

The nomogram demonstrated strong predictive performance for NCR in GH patients following initial RAI therapy, with a C-index of 0.919 (95% CI 0.881-0.957). Calibration curves showed excellent agreement between predicted and observed outcomes (**Figure 5A**). When comparing predictive accuracy, the nomogram (AUC = 0.919) outperformed individual parameters including ΔFT3 (AUC = 0.784), TM (AUC = 0.768), Teff (AUC = 0.753), and RAIU24h (AUC = 0.658) (**Figure 6A**). Additionally, Decision curve analysis confirmed that the nomogram model had good clinical utility in predicting NCR (**Figure 7A**), with validation cohort results mirroring these findings (**Figures 5B**, **6B**, **7B**).

## 4 Discussion

RAI therapy remains a fundamental treatment modality for GH patients, with well-documented efficacy spanning over eight decades of clinical use (5, 7). Contemporary practice patterns reveal a preference among clinicians for administering higher RAI doses (typically 150-200 μCi/g thyroid tissue) to achieve rapid induction of hypothyroidism and reduce the need for retreatment. However, clinical outcomes demonstrate significant variability, with treatment failure rates persisting at 8-15% despite optimized dosimetric approaches, often requiring secondary interventions within 3–6 months of initial RAI therapy (4, 5, 9, 16). While previous investigations have primarily focused on absorbed radiation dose calculations, our study addresses a critical knowledge gap by systematically evaluating four understudied prognostic variables: RAIU24h, Teff, TM, and ΔFT3. Through multivariate analysis of these parameters, we developed and validated a clinically practical nomogram that predicts the probability of NCR following initial RAI therapy, thereby facilitating more personalized therapeutic decision-making.

Our study robustly positions Teff as a significant and independent predictor of treatment failure following RAI therapy for GH disease. The Teff, defined as the time for intra-thyroidal RAI activity to decay to 50% of its initial value, integrates the combined effects of physical decay and biological clearance. It quantifies the composite kinetics of iodine trapping, organification, and retention within thyrocytes (5, 9). Patients exhibiting slower iodine clearance (long Teff) inherently sustain a higher cumulative radiation exposure for any given administered activity per gram compared to those with rapid turnover (short Teff) (17). Consequently, Teff directly determines the cumulative beta-emission absorbed dose; this absorbed dose governs the extent of thyroid follicular cell destruction. While studies by Yu et al. (8) and Wei et al. (18) indicated that a prolonged Teff correlates with a higher incidence of post-therapeutic hypothyroidism, conflicting evidence exists in the literature. Other investigators (19, 20) observed no statistically significant difference in Teff between patients achieving CR and those with NCR following RAI therapy.

Our findings, consistent with prior investigations (9, 21, 22), demonstrate a significant association between elevated RAIU24h and favorable therapeutic outcomes following initial RAI therapy for GH disease. This underscores its fundamental role in thyroidal dosimetry, as RAIU24h directly influences the absorbed radiation dose delivered to the thyroid tissue. However, this positive association contrasts with studies reporting an inverse correlation between RAIU24h and cure rates in GH disease, suggesting reduced efficacy at higher RAIU24h uptake levels (16, 23, 24). This discrepancy may arise from confounding factors inadequately controlled in earlier analyses. Proposed mechanisms include potential inherent radio resistance in thyroid tissue exhibiting higher RAIU24h uptake levels (25), and the critical impact of dosimetry methodology. Within various RAI dosimetric formulae, prescribed RAI activity is typically inversely proportional to RAIU24h values; consequently, subtherapeutic radiation doses derived from such calculations may precipitate treatment failure despite elevated RAIU24h (26, 27), potentially masking the true biological significance of RAIU24h. Notwithstanding these complexities and conflicting reports, our robust statistical analysis controlling for key variables strongly supports the conclusion that RAIU24h constitutes an independent risk factor for RAI therapy efficacy.

Markovic et al. (28) demonstrated significantly superior therapeutic outcomes in GH patients with thyroid glands <62 g (treatment failure rate: 9.6%) compared to those with glands >62 g (persistent hyperthyroidism rate: 44%). This volumetric dependence is further reinforced by Szumowski et al. (29), who demonstrated a significant inverse correlation between thyroid gland volume and therapeutic efficacy (P<0.002), with larger volumes predicting diminished ablative success. Consistent with existing literatures (12, 20, 28, 29), our study confirmed TM as an independent prognostic factor influencing RAI therapy outcomes in GH patients, with increasing TM demonstrating a significant inverse correlation with RAI efficacy. Collectively, these observations substantiate the imperative for rigorous pre-therapeutic assessment in GH patients undergoing RAI therapy, employing quantitative 99mTc-pertechnetate thyroid scan or ultrasonography. This evaluation is critical for accurate glandular volumetry and functional characterization, thereby enabling optimized stratification of the administered RAI activity to achieve therapeutic objectives.

RAI therapy ablates thyroid tissue via β-ray-induced follicular cell necrosis, reducing thyroid hormone synthesis (5). In the present study, the significantly greater reduction in ΔFT3 and ΔFT4 in CR versus NCR groups at 1-month post-therapy reflect the extent of follicular destruction, as FT3 and FT4 kinetics are highly sensitive to thyroidal secretory activity. This phenomenon aligns with GH pathophysiology. Kagayama et al. (30). demonstrated that serum triiodothyronine (T3) concentrations in GH exceed thyroxine (T4) levels by >4-fold, attributable to intrathyroidal preferential T3 secretion—a pathognomonic feature of GH hyperfunction. Consequently, FT3 being independent of thyroxine-binding globulin variations, exhibits superior diagnostic sensitivity for GH. Kaplan et al. (31) further established that accelerated peripheral T4-to-T3 deiodination contributes to elevated FT3 concentrations, while FT3’s 3–4-fold higher biological potency relative to FT4 underscores its critical role in evaluating GH severity. Our multivariate analysis revealed ΔFT3 as an independent predictor of treatment response after adjusting for gender, age, and TRAb levels, confirming its utility in early therapeutic assessment.

The selection of ΔFT3 over ΔFT4 in the final predictive model was guided by the pathophysiology of GH and statistical findings. The characteristic T3-dominant secretory profile of Graves’ hyperthyroidism renders ΔFT3 a more direct biomarker of the early therapeutic effect of RAI on thyroid hormone secretion. Furthermore, in multivariate analysis, ΔFT4 did not retain independent predictive value, indicating that its information was encompassed by other variables, including ΔFT3.

The primary clinical utility of ΔFT3, as identified in our study, lies in early outcome prediction and patient management after the initial RAI therapy. By measuring the magnitude of the FT3 decrease at one month, clinicians can use our nomogram to identify patients with a high probability of requiring additional therapy (e.g., a second RAI treatment or anti-thyroid drugs) at a very early stage. This enables timely clinical decision-making, avoiding prolonged waiting periods to confirm treatment failure based solely on thyroid function tests at 3 or 6 months.

Multivariate logistic regression identified four independent predictors of NCR following initial RAI therapy for GH disease: RAIU24h, Teff, TM, and ΔFT3 (**Table 3**). To enhance prognostic precision beyond individual parameters, we developed a composite nomogram integrating these variables (**Figure 4**). Validation through ROC analysis demonstrated significantly superior discrimination for the nomogram versus any single predictor in both training and validation cohorts (**Figures 6A, B**). The model maintained excellent calibration fidelity and provided positive net benefit across clinical decision thresholds on DCA, supporting its utility for personalized RAI activity prescription. Implementation may optimize therapeutic dosing to reduce retreatment requirements while maintaining remission rates.

However, this study has several limitations requiring acknowledgment. First, the retrospective cohort design inherently carries risks of selection bias. Second, the prognostic nomogram underwent only internal validation without external cohort verification. Third, therapeutic outcomes were assessed solely at short-term follow-up (6–12 months), lacking longitudinal data on relapse rates beyond 24 months. Prospective multicenter studies with larger cohorts are warranted to externally validate this model and evaluate its impact on long-term remission sustainability.

## 5 Conclusion

This study establishes a clinically robust prognostic model for predicting NCR following initial RAI therapy in GH patients. Multivariate analysis identified four independent predictors of treatment failure: TM, RAIU24h, Teff and ΔFT3. The integration of these parameters into a prognostic nomogram demonstrated superior discriminative performance compared to individual variables, with validation cohort results confirming its reliability. Collectively, this nomogram enables personalized RAI activity prescription by stratifying patients at high risk for NCR. Its implementation may optimize therapeutic dosing strategies, reduce retreatment requirements, and improve remission rates.

## Data Availability

The original contributions presented in the study are included in the article/supplementary material. Further inquiries can be directed to the corresponding authors.
